# Characteristics of HIV-Infected Children at Enrollment into Care and at Antiretroviral Therapy Initiation in Central Africa

**DOI:** 10.1371/journal.pone.0169871

**Published:** 2017-01-12

**Authors:** Adebola Adedimeji, Andrew Edmonds, Donald Hoover, Qiuhu Shi, Jean d’Amour Sinayobye, Martin Nduwimana, Patricia Lelo, Denis Nash, Kathryn Anastos, Marcel Yotebieng

**Affiliations:** 1 Department of Epidemiology and Population Health, Albert Einstein College of Medicine, Bronx, New York, United States of America; 2 Department of Epidemiology, The University of North Carolina, Chapel Hill, North Carolina, United States of America; 3 Department of Statistics, Rutgers University, New Brunswick, New Jersey, United States of America; 4 Department of Epidemiology and Community Health, New York Medical College, Valhalla, New York, United States of America; 5 Division of Research and Clinical Education, The Rwanda Military Hospital, Kanombe, Kigali Rwanda; 6 Department of Pediatrics, University Hospital of Kamenge, Faculty of Medicine, University of Burundi, Bujumbura, Burundi; 7 Kalembe Lembe Pediatric Hospital, Kinshasa, The Democratic Republic of Congo; 8 Epidemiology and Biostatistics Program at the City University of New York School of Public Health, New York, New York, United States of America; 9 Department of Medicine, Montefiore Medical Center, Bronx, New York, United States of America; 10 College of Public Health, Division of Epidemiology, The Ohio State University, Columbus, Ohio, United States of America; Katholieke Universiteit Leuven Rega Institute for Medical Research, BELGIUM

## Abstract

**Background:**

Despite the World Health Organization (WHO) regularly updating guidelines to recommend earlier initiation of antiretroviral therapy (ART) in children, timely enrollment into care and initiation of ART in sub-Saharan Africa in children lags behind that of adults. The impact of implementing increasingly less restrictive ART guidelines on ART initiation in Central Africa has not been described.

**Materials and Methods:**

Data are from the Central Africa International Epidemiologic Databases to Evaluate AIDS (IeDEA) pediatric cohort of 3,426 children (0–15 years) entering HIV care at 15 sites in Burundi, DRC, and Rwanda. Measures include CD4 count, WHO clinical stage, age, and weight-for-age Z score (WAZ), each at enrollment into HIV care and at ART initiation. Changes in the medians or proportions of each measure by year of enrollment and year of ART initiation were assessed to capture potential impacts of changing ART guidelines.

**Results:**

Median age at care enrollment decreased from 77.2 months in 2004–05 to 30.3 months in 2012–13. The median age at ART initiation (n = 2058) decreased from 83.0 months in 2004–05 to 66.9 months in 2012–13. The proportion of children ≤24 months of age at enrollment increased from 12.7% in 2004–05 to 46.7% in 2012–13, and from 9.6% in 2004–05 to 24.2% in 2012–13 for ART initiation. The median CD4 count at enrollment into care increased from 563 (IQR: 275, 901) in 2004–05 to 660 (IQR: 339, 1071) cells/μl in 2012–13, and the median CD4 count at ART initiation increased from 310 (IQR:167, 600) in 2004–05 to 589 (IQR: 315, 1113) cells/μl in 2012–13. From 2004–05 to 2012–13, median WAZ improved from -2 (IQR: -3.4, -1.1) to -1 (IQR: -2.5, -0.2) at enrollment in care and from -2 (IQR: -3.8, -1.6) to -1 (IQR: -2.6, -0.4) at ART initiation.

**Discussion and Conclusion:**

Although HIV-infected children ≤24 months of age accounted for half of all children enrolling in care in our cohort during 2012–13, they represented less than a quarter of all those who were initiated on ART during the same period. Further research is needed to identify barriers to timely diagnosis, linkage to care, and initiation of ART among children with HIV infection.

## Introduction

At the end of 2015, an estimated 1.8 million [1.5 million-2.0 million] children less than 15 years of age were living with HIV worldwide, 90% in sub-Saharan Africa [1]. More than 90% of those children acquired their infection through mother-to-child transmission (MTCT) [1]. In the absence of ART, an estimated 26% of postnatally-infected and 52% of perinatally-infected children would die in the first year of HIV infection [2]. Poor immunological, growth, and neurodevelopmental outcomes are common in children who initiate ART at later stages of the disease [3–8].

Access to ART has increased substantially in low- and middle-income countries, with the greatest increase occurring in sub-Saharan Africa: from 100,000 people receiving ART in 2003 to 9.1 million in 2013 [1]. Along with this expansion in ART coverage, the World Health Organization (WHO) explicitly recommends ART initiation in i) all HIV infected children 1–10 years old living with HIV at any CD4 cell count or ii) as a priority among all children <2 years old and those with severe or advanced HIV clinical disease (WHO clinical stage 3 or 4) and individuals with CD4% <25% (if <5 years old) or CD4 count ≤350 cells/mm3 (if ≥5 years old), or iii) among all children <1 year old living with HIV irrespective of CD4 cell count [9, 10]. The impacts of the recommended changes on improved testing coverage (e.g., PMTCT and identification of exposed infants), better infant diagnosis (e.g., PCR) and more importantly immediate initiation of ART among children in Central Africa has not been documented. Although the proportion of HIV-infected children receiving ART in sub-Saharan Africa lags behind that of adults (23% of children vs. 37% of adults in 2013) [1], this disparity is even greater in West and Central Africa, where just 13% of HIV-infected children vs. 32% of adults were receiving ART in 2013 [1].

Only a few published studies have examined temporal trends in the characteristics of children initiating ART in sub-Saharan Africa [11–15]. Although these studies showed a decline over time in disease severity at ART initiation, a substantial proportion of infants and children were still initiating ART at an advanced disease stage [11–13]. To improve timely HIV diagnosis and ART initiation, and to reduce morbidity and mortality among HIV-infected children in resource-constrained settings, it is important to understand the characteristics of children enrolling in care and initiating ART and how these have changed over time as provision of prevention of mother-to-child transmission (improved identification of HIV-exposed infants), early infant diagnosis and testing capacity (e.g., DNA PCR) improves, and HIV treatment guidelines expand in Central Africa. Knowledge of these characteristics will facilitate efforts to identify challenges with timely HIV diagnosis and ART initiation. We thus assessed temporal trends in the characteristics of HIV-infected children at enrollment in HIV care (a proxy for time of diagnosis) and at time of ART initiation from 2004 to 2013 in three Central African countries: Burundi, the Democratic Republic of Congo (DRC), and Rwanda.

## Materials and Methods

### Study setting and participants

We analyzed data on pediatric patients aged 0–15 years from Burundi, the DRC and Rwanda participating in the Central Africa International Epidemiologic Databases to Evaluate AIDS (CA-IeDEA). CA-IeDEA is a multi-country project that collects secondary data from patients receiving HIV care and treatment in the Central African region. The two clinics in the DRC, ten in Rwanda, and three in Burundi that contributed data have been previously described [16–18]. In each participating clinic, data were collected using standardized clinical paper forms that were regularly entered into electronic databases. HIV infection was generally diagnosed by serological testing in children older than 18 months and by virological testing in children younger than 18 months. Children confirmed as HIV-infected by serological or virological testing who were 0–15 years of age at HIV care enrollment between 2004 and 2013 were included in this analysis.

### Variables and statistical analysis

The main variables of interest were CD4 cell count, WHO clinical stage, age, and weight-for-age Z score (WAZ). CD4 count, WAZ and WHO clinical stage at enrollment into care were defined as the measurement taken at or closest to (not later than six months before or after) the enrollment date if no measures were taken on the date of enrollment. CD4 cell counts and WAZ at ART initiation were defined as the measurement taken at ART initiation (i.e. not earlier than 6 months before or later than 3 months after). WAZ was calculated using 2006 WHO standards [19] for children 10 years or younger and Centers for Disease Control and Prevention standards for children older than 10 years [20]. Other continuous variables were categorized as follows: CD4 count (<200, 200–450, >450 cells/μl), and age (0–24, 25–60, 61–84, 85–120, and 121–156 months). Year of enrollment and year of ART initiation were grouped as 2004–05, 2006–07, 2008–09, 2010–11, and 2012–13 to reflect temporal changes in WHO guidelines over time.

Demographic characteristics (age and sex) and HIV-associated characteristics (CD4 count, WHO stage, and WAZ score) at care enrollment and ART initiation were analyzed. Descriptive statistics (numbers and percentages for categorical variables, and median and interquartile range (IQR) for continuous variables) were calculated. Chi-square and Kruskal-Wallis tests were used to examine changes in distributions over the 10-year period.

Ethical approval for the study was granted by the Albert Einstein College of Medicine Institutional Review Board in New York, and by the relevant ethics review boards in Rwanda (National Health Research Committee and National Ethics Committee), Burundi (Comite National d’ Ethique) and DRC (Ministère de l'enseignement supérieur et universitaire, Université de Kinshasa École de santé publique). Data for the Central Africa International Epidemiologic Database to Evaluate AIDS (CA-IeDEA) is publicly available upon request as part of the global IeDEA consortium.

## Results

Table 1 shows characteristics of children when they enrolled into care (n = 3426) and at ART initiation (n = 2058). Overall, from 2004–13, the median age at enrollment was 58.2 months (interquartile range (IQR); 19.8, 102) and 70.3 (IQR; 29.2, 113) at ART initiation. Nearly equal proportions of boys and girls enrolled in care, and initiated ART over the 10-year period.

**Table 1:** Characteristics of children at enrollment in care and at ART initiation in the Central Africa IeDEA Cohort, 2004–2013.

	Enrollment	ART Initiation
	n = 3426 (%)	n = 2058 (%)
**Age (months) (n, %)**		
Median (IQR)	58.2 (19.8; 102)	70.3 (29.2; 113)
0–24	982 (28.7)	422 (20.5)
25–60	775 (22.6)	497 (24.1)
61–84	489 (14.3)	267 (13.0)
85–120	626 (18.3)	439 (21.3)
121–156	554 (16.2)	433 (21.0)
**Sex (n, %)**		
Male	1669 (48.7)	1047 (50.9)
Female	1757 (51.3	1011 (49.1)
**Country (n, %)**		
Burundi	722 (21.1)	299 (14.5)
DRC	1307 (38.1)	1002 (48.7)
Rwanda	1397 (40.8)	757 (36.8)
**CD4 Count (n, %)**		
Median (IQR)	648 (339; 1029)	457 (249; 936)
<200	309 (14.2)	325 (19.6)
200–450	429 (19.7)	493 (29.7)
>450	1440 (66.1)	842 (50.7)
**WHO Clinical Stage (n, %)**		
No Data	1185 (34.6)	335 (16.3)
I	611 (17.8)	310 (15.1)
II	625 (18.2)	411 (20.0)
III	901 (26.3)	903 (43.9)
IV	104 (3.0)	99 (4.8)
**Weight-for-age Z-score**		
Median (IQR)	-1.8 (-3.0;-0.7)	-1.9 (-3.0; -0.8)
>-2	1276 (55.1)	922 (52.9)
-3 to -2	455 (19.8)	377 (21.6)
<3	585 (25.3)	445 (25.5)
**Year (n, %)**		
2004–05	630 (18.4)	280 (13.6)
2006–07	680 (19.8)	431 (20.9)
2008–09	697 (20.3)	413 (20.1)
2010–11	729 (21.3)	468 (22.7)
2012–13	690 (20.1)	466 (22.6)

The median time from enrollment in care to ART initiation for the different age groups are: 1.3 for 0–24 and 25–60 months, 2.1 for 61–84 months, 3.1 for 85–120 months and 2.4 for 121–156 months. The median CD4 count was 648 cells/μl (IQR; 339, 1029) at enrollment into care and 457 cells/μl (IQR; 249, 936) at ART initiation. WHO clinical staging data were not available for more than one-third of children at care enrollment and for about 16% of children at ART initiation. Over one-third of children with WHO staging data were classified as stage III or IV when they enrolled into care, with nearly half (48%) of these classified as stage III or IV at ART initiation. Median WAZ was -1 (IQR; -3.0, -0.7) at care enrollment and -1 (IQR; -3.0, 0.8) at ART initiation. Overall, children ≤24 months of age were 29% of those enrolled in care and 21% of those initiated on ART.

Table 2 reports children’s characteristics by year of enrollment into care. Median age at enrollment fell from 77.2 months in 2004–05 to 30.3 months in 2012–13 (p = <0.0001). The proportion of children ≤24 months of age who enrolled in care increased from 13% in 2004–05 to 47% in 2012–13. Median CD4 count at enrollment in care increased from 563 in 2004–05 to 660 cells/μl in 2012–13 (p = <0.0001). The proportion of children with CD4 count <200 cells/μl and 200–349 cells/μl also declined from 2004–05 to 2012–13 but remained fairly stable among those whose CD4 count is >350–499 cells/μl and >500 cells/μl. Median CD4 count increased marginally in all age groups, with the highest increase among children 0–24 months (p = 0.04) and lowest among those aged 121–156 months (p = 0.26). The proportion of children classified as stage III or IV at enrollment decreased from 37% in 2004–05 to 20% in 2012–13. Median WAZ was -2 in 2004–05 and -1 in 2012–13 (p = <0.0001).

**Table 2:** Characteristics of children in the Central Africa IeDEA cohort by year of enrollment.

	2004–05	2006–07	2008–09	2010–11	2012–13	*p*-value
	n = 630 (%)	n = 680 (%)	n = 697 (%)	n = 729 (%)	n = 690 (%)	
**Age (months) (n, %)**						
Median (IQR)	77.2(44.8,110)	74.5(35.6,111)	53.8(21.0,98.0)	40.5(12.3,90.7)	30.3(5.9,87.6)	<0.0001
0–24	80 (12.7)	114 (16.8)	195 (28.0)	272 (37.3)	321 (46.5)	<0.0001
25–60	151 (24.0)	157 (23.1)	178 (25.5)	165 (22.6)	124 (18.0)	
61–84	126 (20.0)	118 (17.4)	98 (14.1)	83 (11.4)	64 (9.3)	
85–120	155 (24.6)	155 (22.8)	119 (17.1)	108 (14.8)	89 (12.9)	
121–156	118 (18.7)	136 (20.0)	107 (15.4)	101 (13.9)	92 (13.3)	
**Sex (n,%)**						
Male	291 (46.2)	337 (49.6)	360 (51.6)	344 (47.2)	337 (48.8)	0.3
Female	339 (53.8)	343 (50.4)	337 (48.4)	385 (52.8)	353 (51.2)	
**Country (n,%)**						
Burundi	NA	NA	194 (27.8)	277 (38.0)	251 (36.4)	<0.0001
DRC	268 (42.5)	256 (37.6)	289 (41.5)	291 (39.9)	203 (29.4)	
Rwanda	362 (57.5)	424 (62.4)	214 (30.7)	161 (22.1)	236 (34.2)	
**CD4 count (n, %)**						
Missing Data (Not in p-value)	93 (14.8)	91 (13.4)	282 (40.5)	330 (45.3)	452 (65.5)	<0.0001
Overall Median (IQR)	563(275,901)	670(361,1056)	675(349,1104)	695(398,1084)	660(339,1071)	0.001
<200	96 (17.9)	75 (12.7)	56 (13.5)	53 (13.3)	29 (12.2)	
200–349	81 (15.1)	63 (10.7)	48 (11.6)	31 (7.8)	31 (13.0)	
>350–499	64 (11.9)	82 (13.9)	45 (10.8)	47 (11.8)	20 (8.4)	
>500	296 (55.1)	369 (62.6)	266 (64.1)	268 (67.2)	158 (66.4)	
**Median CD4 count by age (IQR)**						
0–24 month	995 (542, 1629)	1240 (923, 2009	1176 (675, 1912)	1066 (667, 1695)	1012 (672, 1664)	0.04
25–60 months	778 (395, 1040)	760 (491, 1207)	842 (475, 1137)	779 (527, 1224)	851 (596, 1161)	0.55
61–84 months	555 (338, 832)	685 (351, 1003)	635 (276, 974)	633 (397, 920)	671 (439, 857)	0.61
85–120 months	424 (201, 669)	480 (277, 828)	500 (270, 823)	499 (247, 840)	441 (214, 686)	0.41
121–156 months	331 (181, 577)	397 (235, 692)	366 (169, 612)	370 (108, 704)	505 (298, 871)	0.26
n for 0–24 months	63	92	89	102	46	
n for 25–60 months	129	144	113	108	56	
n for 61–84 months	112	105	68	56	33	
n for 85–120 months	134	135	74	65	50	
n for 121–156 months	99	113	71	68	53	
**WHO clinical stage (n,%)**						
Missing data (Not in p-value)	233 (37.0)	260 (38.2)	215 (30.8)	240 (32.9)	237 (34.4)	<0.0001
I	57 (9.0)	70 (10.3)	129 (18.5)	152 (20.9)	203 (29.4)	
II	108 (17.1)	135 (19.9)	130 (18.6)	140 (19.2)	112 (16.2)	
III	206 (32.7)	200 (29.4)	200 (28.7)	172 (23.6)	123 (17.8)	
IV	26 (4.1)	15 (2.2)	23 (3.3)	25 (3.4)	15 (2.2)	
**Weight-for-age Z-score (n,%)**						
Missing data (Not in p-value)	220 (34.9)	255 (37.5)	178 (25.5)	235 (32.2)	222 (32.2)	
Median (IQR)	-2.2(-3.4,-1.1)	-1.9(-3.2,-0.8)	-1.6 (-2.9–0.6)	-1.7(-3.1,-0.5)	-1.4(-2.5,-0.2)	<0.0001
>-2	181 (44.1)	220 (51.8)	299 (57.6)	277 (56.1)	299 (63.9)	<0.0001
-3 to -2	106 (25.9)	79 (18.6)	98 (18.9)	84 (17.0)	88 (18.8)	
<-3	123 (30.0)	126 (29.6)	122 (23.5)	133 (26.9)	81 (17.3)	

Characteristics of children at care enrollment by country are shown in Tables 3–5. In Burundi, the median age decreased from 53.5 months in 2008–09 to 33.0 months in 2010–11, and then increased to 49.7 months in 2012–13. In the DRC, a similar trend was observed with the median age decreasing from 76.8 months in 2004–05 to 40.7 months in 2012–13. However, in Rwanda, though the median age in 2004–05 and 2006–07 was similar to that observed in DRC, it decreased continuously to 11.4 months in 2012–13. The proportion of children ≤24 months of age mostly drove the decreasing trend in median age at enrollment across countries. Between 2010–11 and 2012–13, this proportion more than doubled from 32.3% to 66.1% in Rwanda, changed slightly in DRC (from 35.0% to 37.7%), but decreased in Burundi, from 43% to 36%.

**Table 3:** Characteristics at enrollment in care of children in Central Africa IeDEA in Burundi by year of enrollment.

	2004–05 (n, %)	2006–07 (n,%)	2008–09 n = 194 (%)	2010–11 n = 277 (%)	2012–13 n = 251 (%)	*p*-Value
**Age (months)(n,%)**						
Median (IQR)			53.5 (18.5, 98.4)	33.0 (8.1, 93.5)	49.7 (11.6, 97.4)	0.19
0–24			55 (28.3)	118 (42.6)	90 (35.9)	0.17
25–60			51 (26.3)	53 (19.1)	50 (19.9)	
61–84			23 (11.9)	27 (9.6)	27 (10.8)	
85–120			35 (18.0)	40 (14.4)	42 (16.7)	
121–156			30 (15.5)	39 (14.1)	42 (16.7)	
**Sex (n,%)**						
Male			99 (51.0)	123 (44.4)	118 (47.0)	0.36
Female			95 (49.0)	154 (55.6)	133 (53.0)	
**CD4 count (n,%)**						
Missing Data (Not in P-value)			152 (78.4)	217 (78.34)	187 (74.5)	
Median (Q1,Q3)			712(512,1124)	562(347,988)	698(418,978)	0.25
<200			5 (11.9)	8 (13.3)	4 (6.3)	0.03
200–349				7 (11.7)	7 (10.9)	
350–499			4 (9.5)	11 (18.3)	6 (9.4)	
>500			33 (78.6)	34 (56.7)	47 (73.4)	
Median CD4 for 0–24 months			1092(750, 2010)	828(491,1427)	1143(518,1978)	0.61
Median CD4 for 25–60 months			987(536,1299)	843(386,1375)	943(618,1818)	0.64
Median CD4 for 61–84 months			640(525,754)	446(315,985)	597(504,809)	0.76
Median CD4 for 85–120 months			911(599,1178)	513(379,619)	582(312,775)	0.14
Median CD4 for 121–156 months			497(279,638)	342(151,577)	616(408,924)	0.06
n for 0–24 months			4	15	10	
n for 25–60 months			12	17	11	
n for 61–84 months			9	6	11	
n for 85–120 months			9	8	16	
n for 121–156 months			8	14	16	
**WHO clinical stage (n,%)**						
Missing Data (Not in P-value)			92 (47.4)	147 (53.1)	56 (22.3)	
Stage I			19 (18.6)	21 (16.2)	58 (29.7)	0.047
Stage II			24 (23.5)	41 (31.5)	47 (24.1)	
Stage III			46 (45.1)	55 (42.3)	77 (39.5)	
Stage IV			13 (12.7)	13 (10.0)	13 (6.7)	
**Weight-for-age Z-score (n,%)**						
Missing Data (Not in P-value)			97 (50.0)	160 (57.8)	57 (22.7)	
Median (Q1, Q3)			-1.4(-2.3,-0.2)	-1.6(-3.4,-0.2)	-1.3(-2.5,0.3)	0.077
>-2			67 (69.1)	68 (58.1)	123 (63.4)	0.035
-3 to -2			10 (10.3)	15 (12.8)	38 (19.6)	
<-3			20 (20.6)	34 (29.1)	33 (17.0)	

**Table 4:** Characteristics at enrollment in care of children in Central Africa IeDEA in DRC by year of enrollment.

	2004–05 n = 268 (%)	2006–07 n = 256 (%)	2008–09 n = 289 (%)	2010–11 n = 291 (%)	2012–13 n = 203 (%)	*p*-Value
**Age (months) (n,%)**						
Median (IQR)	76.8 (43.3, 114)	66.1(29.8,103)	49.8 (16.3, 85.1)	42.4(15.4,86.8)	40.7(8.2,96.6)	<0.0001
0–24	35 (13.1)	53 (20.7)	96 (33.3)	102 (35.1)	75 (36.9)	<0.0001
25–60	68 (25.4)	66 (25.8)	74 (25.0)	75 (25.8)	49 (24.1)	
61–84	52 (19.4)	42 (16.4)	41 (14.2)	38 (13.1)	21 (10.3)	
85–120	61 (22.8)	51 (19.9)	42 (14.5)	43 (14.8)	28 (13.8)	
121–156	52 (19.4)	44 (17.2)	33 (11.0)	33 (11.3)	30 (14.8)	
**Sex (n,%)**						
Male	123 (45.9)	125 (48.8)	144 (49.8)	149 (51.2)	105 (51.7)	0.7
Female	145 (54.1)	131 (51.2)	145 (50.2)	142 (48.8)	98 (48.3)	
**CD4 count (n,%)**						
Missing Data (Not in P-value)	11 (4.1)	17 (6.6)	90 (31.1)	62 (21.3)	102 (50.3)	
Median (Q1,Q3)	543(253,852)	631(273,1076)	618(293,1011)	656(364,1066)	610(316,1017)	0.09
<200	53 (20.6)	46 (19.2)	36 (18.1)	40 (17.5)	15 (14.9)	0.32
200–349	31 (12.1)	20 (8.4)	26 (13.1)	16 (7.0)	13 (12.9)	
350–499	38 (14.8)	29 (12.1)	21 (10.6)	27 (11.8)	9 (8.9)	
>500	135 (52.5)	144 (60.3)	116 (58.3)	146 (63.8)	64 (63.4)	
Median CD4 for 0–24 months	1191(706,1757)	1204(765,1846)	1029(616,1912)	1066(576,1670)	1133(672,1292)	0.8
Median CD4 for 25–60 months	607(275,942)	668(451,1096)	719(359,988)	759(448,1057)	640(511,1017)	0.44
Median CD4 for 61–84 months	495(331,802)	668(199,976)	389(116,900)	618(438,855)	680(386,1285)	0.22
Median CD4 for 85–120 months	431(188,649)	442(249,907)	377(195,627)	387(130,642)	431(252,612)	0.68
Median CD4 for 121–156 months	317(133,577)	257(49,585)	295(100,579)	151(25,442)	365(113,547)	0.17
n for 0–24 months	35	48	56	68	18	
n for 25–60 months	63	64	58	64	31	
n for 61–84 months	52	41	31	34	12	
n for 85–120 months	57	48	26	35	19	
n for 121–156 months	50	38	28	28	21	
**WHO clinical stage (n,%)**						
Missing Data (Not in P-value)	3 (1.1)	14 (5.5)	22 (7.6)	10 (3.4)	12 (5.9)	
Stage I	29 (10.9)	40 (16.5)	71 (26.6)	83 (29.5)	109 (57.1)	<0.0001
Stage II	65 (24.5)	71 (29.3)	68 (25.5)	84 (29.9)	42 (22.0)	
Stage III	151 (57.0)	119 (49.2)	122 (45.7)	106 (37.7)	39 (20.4)	
Stage IV	20 (7.5)	12 (5.0)	6 (2.2)	8 (2.8)	1 (0.5)	
**Weight-for-age Z-score(n,%)**						
Missing Data (Not in P-value)	3 (1.1)	3 (1.2)	2 (0.7)	1 (0.3)	5 (2.5)	
Median (Q1, Q3)	-2.4(-3.6,-1.3)	-2.1(-3.3,-1.0)	-1.9(-3.2,-0.7)	-2.1(-3.3,-0.9)	-1.4(-2.7,-0.5)	<0.0001
>-2	106 (40.0)	119 (47.0)	147 (51.2)	141 (48.6)	127 (64.1)	0.0003
-3 to -2	69 (26.0)	51 (20.2)	60 (20.9)	58 (20.0)	32 (16.2)	
<-3	90 (34.0)	83 (32.8)	80 (27.9)	91 (31.4)	39 (19.7)	

**Table 5:** Characteristics at enrollment in care of children in Central Africa IeDEA in Rwanda by year of enrollment.

	2004–05 n = 362 (%)	2006–07 n = 424 (%)	2008–09 n = 214 (%)	2010–11 n = 161 (%)	2012–13 n = 236 (%)	*p*-Value
**Age (months) (n,%)**						
Median (IQR)	77.6 (45.6, 109)	78.4 (41.0, 116)	68.5 (27.7, 109)	50.7 (15.2, 97.0)	11.4 (2.2, 49.7)	<0.0001
0–24	45 (12.4)	61 (14.4)	44 (20.6)	52 (32.3)	156 (66.1)	<0.0001
25–60	83 (22.9)	91 (21.5)	53 (24.8)	37 (23.0)	25 (10.6)	
61–84	74 (20.4)	76 (17.9)	34 (15.9)	18 (11.2)	16 (6.8)	
85–120	94 (26.0)	104 (24.5)	42 (19.6)	25 (15.5)	19 (8.0)	
121–156	66 (18.2)	92 (21.1)	41 (19.2)	29 (18.0)	20 (8.5)	
**Sex (n,%)**						
Male	168 (46.4)	212 (50.0)	117 (54.7)	72 (44.7)	114 (48.3)	0.28
Female	194 (53.6)	212 (50.0)	97 (45.3)	89 (55.3)	122 (51.7)	
**CD4 count (n,%)**						
Missing Data (Not in P-value)	82 (22.7)	74 (17.5)	40 (18.7)	51 (31.7)	163 (69.1)	<0.0001
Median (Q1,Q3)	582(298,927)	681(377,1026)	736(376,1159)	801(598,1213)	741(301,1123)	0.0005
<200	43 (15.4)	29 (8.3)	15 (8.6)	5 (4.5)	10 (13.7)	
200–349	50 (17.9)	43 (12.3)	22 (12.6)	8 (7.3)	11 (15.1)	
350–499	26 (9.3)	53 (5.1)	20 (11.5)	9 (8.2)	5 (6.8)	
>500	161 (57.5)	225 (64.3)	117 (67.2)	88 (80.0)	47 (64.4)	
Median CD4 for 0–24 months	800(259,1411)	1344(1014,2143)	1305(1106,1912)	1240(965,1971)	951(698,1559)	0.0075
Median CD4 for 25–60 months	882(548,1199)	855(555,1254)	999(620,1213)	1000(665,1335)	983(696,1216)	0.5746
Median CD4 for 61–84 months	648(390,934)	695(399,1016)	760(476,1266)	738(400,1017)	709(387,981)	0.6195
Median CD4 for 85–120 months	378(212,727)	498(307,744)	461(302,819)	856(499,1117)	305(135,938)	0.0066
Median CD4 for 121–156 months	345(224,568)	460(273,755)	385(254,612)	675(362,796)	586(301,1132)	0.0415
n for 0–24 months	28	44	29	19	18	
n for 25–60 months	66	80	43	27	14	
n for 61–84 months	60	64	28	16	10	
n for 85–120 months	77	87	39	22	15	
n for 121–156 months	49	75	35	26	16	
**WHO clinical stage (n,%)**						
Missing Data (Not in P-value)	230 (63.5)	246 (58.0)	101 (47.2)	83 51.6)	169 (71.6)	
Stage I	28 (21.2)	30 (16.9)	39 (34.5)	48 (61.5)	36 (53.7)	0.0001
Stage II	43 (32.6)	64 (36.0)	38 (33.6)	15 (19.2)	23 (34.3)	
Stage III	55 (41.7)	81 (45.5)	32 (28.3)	11 (14.1)	7 (10.4)	
Stage IV	6 (4.5)	3 (1.7)	4 (3.5)	4 (5.1)	1 (1.5)	
**Weight-for-age Z-score (n,%)**						
Missing Data (Not in P-value)	217 (59.9)	252 (59.4)	79 (36.9)	74 (46.0)	160 (67.8)	
Median (IQR)	-1.8(-2.9,-0.9)	-1.7(-3.0,-0.6)	-1.4(-2.4,-0.6)	-0.9(-1.7,-0.1)	-1.6(-2.3,-0.4)	0.001
>-2	75 (51.7)	101 (58.7)	85 (63.0)	68 (78.2)	49 (64.5)	0.002
-3 to -2	37 (25.5)	28 (16.3)	28 (20.7)	11 (12.6)	18 (23.7)	
<-3	33 (22.8)	43 (25.0)	22 (16.3)	8 (9.2)	9 (11.8)	

The proportion of children with WHO staging data who were classified as stage III or IV at enrollment in care in Burundi was 30%, 25% and 36% in 2008–09, 2010–11, and 2012–13, respectively. In the DRC, the proportion of children classified as stage III or IV was 64% in 2004–05 and decreased continuously to 20% in 2012–13, whereas the proportion in Rwanda decreased from a peak of 19% in 2006–2007 to 16% in 2008–09, 9.3% in 2010–11, and 3.4% in 2012–13. Nearly equal proportions of boys and girls enrolled in care in each country from 2004–13. Median WAZ increased progressively from 2004–13 in the DRC and Rwanda, but fluctuated in Burundi from 2008–09 to 2012–13.

Table 6 shows children’s characteristics at ART initiation by year, from 2004 to 2013. The median age at ART initiation was 83.0 months in 2004–05, progressively decreased until 2010–11 to a median of 49.2 months, and then rose to 67.2 months in 2012–13 (p = <0.0001). The proportion of ART initiators ≤24 months of age increased from 9.6% in 2004–05 to a peak of 29.2% in 2010–11, before declining to 24.2% in 2012–13. During the same period, the median CD4 count increased from 310 cells/μl in 2004–05 to 589 cells/μl in 2012–13 (p = <0.0001). The proportion of children with median CD4 count <200 at ART initiation decreased from 32% in 2004–05 to 13% in 2012–13 whereas it fluctuated or remained fairly stable among children with >200 cells/μl. Median CD4 count at ART initiation increased among all age groups from 2004–05 to 2012–13, with the most significant increase observed among children aged 25–60 months (p = 0.0003).

**Table 6:** Characteristics of children in the Central Africa IeDEA cohort by year of ART initiation.

	2004–05 n = 280 (%)	2006–07 n = 431 (%)	2008–09 n = 413 (%)	2010–11 n = 468 (%)	2012–13 n = 466 (%)	*p*-value
**Age (months) (n, %)**						
Median (IQR)	83.0 (49.8, 117)	79.3 (38.5, 117)	71.3 (27.8, 112)	49.9(21.7,106)	67.2(25.2,115)	<0.0001
0–24	27 (9.6)	65 (15.1)	87 (21.1)	133 (28.42)	110 (23.6)	<0.0001
25–60	66 (23.6)	102 (23.7)	94 (22.8)	127 (27.14)	108 (23.2)	
61–84	52 (18.6)	63 (14.3)	55 (13.3)	45 (9.62)	52 (11.2)	
85–120	73 (26.1)	103 (23.9)	94 (22.8)	79 (16.8)	90 (19.3)	
121–156	62 (22.1)	98 (22.7)	83 (20.1)	84 (17.95)	106 (22.7)	
**Sex (n,%)**						
Male	136 (48.6)	227 (52.7)	220 (53.3)	239 (51.07%)	230 (49.4)	0.73
Female	144 (51.4)	204 (47.3)	193 (46.3)	229 (48.93%)	236 (50.6)	
**Country (n,%)**						
Burundi	N/A	N/A	28 (6.8)	63 (13.5)	208 (44.6)	<0.0001
DRC	165 (58.9)	227 (52.7)	214 (51.8)	265 (56.6)	131 (28.1)	
Rwanda	115 (41.1)	204 (47.3)	171 (41.4)	140 (29.9)	127 (27.2)	
**CD4 count (n, %)**						
Missing Data (Not in p-value)	23 (8.2)	29 (6.7)	71 (17.2)	90 (19.2)	185 (39.7)	
Median (IQR)	310 (167, 600)	471 (228, 950)	437 (246, 879)	560 (317,1066)	589 (315,1113)	
<200	83 (32.3)	85 (21.1)	62 (18.1)	58 (15.3)	37 (13.17)	<0.0001
200–349	62 (24.1)	73 (18.2)	84 (24.6)	57 (15.1)	58 (20.6)	<0.0001
350–499	35 (13.6)	54 (13.4)	39 (11.4)	57 (15.1)	32 (11.4)	
>500	77 (30.0)	190 (47.3)	157 (45.9)	206 (54.5)	154 (54.1)	
**Median by age group (IQR)**						
0–24 months	883 (611, 1291)	1140 (832, 1784)	1073 (684, 1784)	1158 (705, 1796)	1267 (845, 2141)	0.06
25–60 months	469 (240, 838)	628 (425, 1029)	726 (360, 1042)	703 (435, 1151)	_889 (610, 1358)	0.0003
61–84 months	332 (178, 482)	544 (264, 867)	432 (227, 697)	546 (329, 684)	597 (329, 655)	0.01
85–120 months	219 (120, 378)	339 (204, 615)	302 (214, 419)	327 (174, 462)	338 (217, 655)	0.003
121–156 months	223 (109, 321)	249 (131, 424)	267 (169, 358)	315 (97, 428)	342 (246, 470)	0.01
n for 0–24 months	27	62	66	102	60	
n for 25–60 months	58	94	79	102	57	
n for 61–84 months	49	61	47	38	33	
n for 85–120 months	67	95	81	64	63	
n for 121–156 months	56	90	69	72	68	
**WHO clinical stage (n,%)**						
Missing data (Not in p-value)	68 (24.3)	71 (16.5)	70 (16.9)	64 (13.68%)	62 (13.3)	
I	17 (6.1)	45 (10.4)	58 (14.0)	81 (17.31%)	109 (23.4)	<0.0001
II	40 (14.3)	91 (21.1)	88 (21.3)	93 (19.87%)	99 (21.2)	
III	136 (48.6)	206 (47.8)	183 (44.3)	199 (42.52%)	179 (38.4)	
IV	19 (6.8)	18 (4.2)	14 (3.4)	31 (6.62%)	17 (3.6)	
**Weight-for-age Z-score (n, %)**						
Missing data (Not in p-value)	71	88	60	62	33	
Median (IQR)	-2.6 (-3.8, -1.6)	-2.0 (-3.2, 0.9)	-2.0 (-.3.0, -0.9)	-1.8(-3.0,-0.7)	-1.5(-2.6,-0.4)	<0.0001
>-2	70 (33.5)	176 (51.3)	181 (51.3)	221 (54.4)	274 (63.3)	<0.0001
-3 to -2	53 (25.4)	70 (20.4)	83 (23.5)	85 (20.9)	86 (19.9)	
<-3	86 (41.1)	97 (28.3)	89 (25.2)	100 (24.6)	73 (16.9)	

The overall proportion of children without WHO clinical staging data declined from 24% in 2004–05 to about 13% in 2012–13. Among children with WHO staging data reported, the proportion of those classified as stage III or IV decreased from 54% 2004–05 to 42% to 2012–13 (p = <0.0001). There was improvement in the median WAZ, from -2 in 2004–05 to -1 in 2012–13 (p = <0.0001).

Tables 7–9 depict children’s characteristics at ART initiation in each country. In Burundi, the median age was 71.4 months in 2008–09 but remained fairly stable from 2010–13. The proportion of children ≤24 months of age who initiated ART from 2008–09 to 2012–13 increased from 11% to 16%. In the DRC, the median age remained stable at ~75 months between 2004–07 then declined to 40.2 months in 2010–11 before rising to 59.9 months in 2012–13. The proportion of ART initiators ≤24 months of age increased from 11.5% in 2004–05 to 33% in 2010–11, before dropping to 24% in 2012–13. In Rwanda, the median age at ART initiation declined from 90.2 months in 2004–05 to 44.8 months in 2012–13, driven by the increasing proportion of children ≤24 months of age, which rose from 7% to 35% over the same period. The median CD4 count increased by about 200 and 300 cells/μl in the DRC and Rwanda respectively between 2004–05 and 2012–13, and by more than 100 cells/μl in Burundi from 2008–09 to 2012–13. The proportion of children classified as WHO clinical stage III or IV in each country was noteworthy. Nearly half and slightly more than half of children in Burundi and the DRC, respectively, and nearly one-third of children in Rwanda were classified as stage III or IV. Considerable proportions of children in the three countries were underweight at ART initiation, although these proportions continuously declined from 2004-05-2012-13.

**Table 7:** Characteristics at ART initiation of children in Central Africa IeDEA in Burundi by year of ART initiation.

	2004–05 (n, %)	2006–07 (n, %)	2008–09 n = 28 (%)	2010–11 n = 63 (%)	2012–13 n = 208 (%)	*p*-Value
**Age (months) (n,%)**						
Median (IQR)			71.4 (41.0, 124)	80.4 (32.6, 123)	79.1 (35.9, 123)	0.97
0–24			3 (10.7)	10 (15.9)	34 (16.3)	0.89
25–60			8 (28.6)	18 (28.6)	48 (23.1)	
61–84			5 (17.9)	7 (11.1)	27 (13.0)	
85–120			3 (10.7)	11 (17.5)	43 (20.7)	
121–156			9 (32.1)	17 (27.0)	56 (26.9)	
**Sex (n,%)**						
Male			16 (57.1)	34 (54.0)	102 (49.0)	0.62
Female			12 (42.9)	29 (46.0)	106 (51.0)	
**CD4 count (n,%)**						
Missing Data (Not in P-value)			20 (71.4)	31 (49.2)	130 (62.5)	
Median (Q1,Q3)			519(168,662)	383(215,599)	644(376,1080)	0.002
<200			3 (37.5)	7 (21.9)	7 (9.0)	0.013
200–349				5 (15.6)	9 (11.5)	
350–499				11 (34.4)	16 (20.5)	
>500			5 (62.5)	9 (28.1)	46 (59.0)	
Median CD4 for 0–24 months				623(447,828)	565(293,1934)	0.8383
Median CD4 for 25–60 months			632(163,1100)	419(358,637)	736(618,1190)	0.1797
Median CD4 for 61–84 months			603(511,695)	333(264,392)	840(418,1225)	0.0882
Median CD4 for 85–120 months			526(526,526)	389(155,493)	644(411,1190)	0.033
Median CD4 for 121–156 months			173(151,629)	325(132,575)	416(343,780)	0.2182
n for 0–24 months				3	8	
n for 25–60 months			2	8	13	
n for 61–84 months			2	4	13	
n for 85–120 months			1	7	22	
n for 121–156 months			3	10	22	
**WHO clinical stage (n,%)**						
Missing Data (Not in P-value)			16 (57.1)	21 (33.3)	17 (8.2)	
Stage I				1 (2.38)	27 (14.1)	0.049
Stage II				8 (19.05)	33 (17.3)	
Stage III			10 (83.3)	32 (76.19)	116 (60.7)	
Stage IV			2 (16.7)	1 (2.38)	15 (7.9)	
**Weight-for-age Z-score(n,%)**						
Missing Data (Not in P-value)			18 (64.3)	30 (47.6)	16 (7.7)	
Median (Q1, Q3)			-1.0(-1.6,-0.6)	-2.3(-3.2,-1.3)	-1.5(-2.7,-0.4)	0.07
>-2			9 (90.0)	16 (48.5)	120 (62.5)	0.16
-3 to -2			1 (10.0)	7 (21.2)	35 (18.2)	
<-3				10 (30.3)	37 (19.3)	

**Table 8:** Characteristics at ART initiation of children in Central Africa IeDEA in the DRC by year of ART initiation.

	2004–05 n = 165 (%)	2006–07 n = 227 (%)	2008–09 n = 214 (%)	2010–11 n = 265 (%)	2012–13 n = 131 (%)	*p*-Value
**Age (months) (n,%)**						
Median (IQR)	74.8 (45.6, 109)	74.9 (35.9, 113)	54.3 (21.9, 96.4)	40.2(18.5,84.4)	59.9(25.2,111)	<0.0001
0–24	19 (11.5)	36 (15.9)	61 (28.5)	87 (32.8)	31 (23.7)	<0.0001
25–60	44 (26.7)	57 (25.2)	54 (25.2)	83 (31.3)	35 (26.7)	
61–84	31 (18.8)	37 (16.3)	30 (14.0)	26 (9.8)	14 (10.7)	
85–120	38 (23.0)	47 (20.7)	43 (20.1)	34 (12.8)	25 (19.1)	
121–156	33 (20.0)	50 (22.0)	26 (12.1)	35 (13.2)	26 (19.8)	
**Sex (n,%)**						
Male	80 (48.5)	108 (47.6)	110 (51.4)	136 (51.3)	67 (51.1)	0.9
Female	85 (51.5)	119 (52.4)	104 (48.6)	129 (48.7)	64 (48.9)	
**CD4 count (n,%)**						
Missing Data (Not in P-value)	8 (4.9)	8 (3.5)	36 (16.8)	42 (15.9)	29 (22.1)	
Median (Q1,Q3)	359(162,675)	552(218,1015)	545(233,911)	656(317,1109)	547(278,963)	<0.0001
<200	48 (30.6)	50 (22.8)	39 (21.9)	40 (17.9)	18 (17.7)	0.0002
200–349	29 (18.5)	26 (11.9)	26 (14.6)	24 (10.8)	21 (20.6)	
350–499	22 (14.0)	30 (13.7)	21 (11.8)	26 (11.7)	9 (8.8)	
>500	58 (36.9)	113 (51.6)	92 (51.7)	133 (59.6)	54 (52.9)	
Median CD4 for 0–24 months	838(667,1282)	1108(757,1827)	888(655,1430)	1117(667,1774)	1237(845,2047)	0.1543
Median CD4 for 25–60 months	443(215,892)	630(389,1061)	708(347,984)	758(428,1067)	640(511,1029)	0.152
Median CD4 for 61–84 months	335(189,574)	667(125,931)	378(211,697)	591(349,737)	439(319,601)	0.2452
Median CD4 for 85–120 months	224(68.0,491)	456(233,866)	266(158,533)	267(102,474)	262(87.0,337)	0.0902
Median CD4 for 121–156 months	237(116,377)	236(60.0,438)	169(41.0,484)	115(28.0,360)	310(133,370)	0.4592
n for 0–24 months	19	35	45	71	24	
n for 25–60 months	39	57	48	71	25	
n for 61–84 months	30	37	26	22	9	
n for 85–120 months	36	44	36	28	20	
n for 121–156 months	33	46	23	31	24	
**WHO clinical stage (n,%)**						
Missing Data (Not in P-value)					1 (0.8)	<0.0001
Stage I	9 (5.5)	28 (12.3)	25 (11.7)	49 (18.5)	41 (31.5)	
Stage II	27 (16.4)	60 (26.4)	62 (29.0)	62 (23.4)	41 (31.5)	
Stage III	115 (69.7)	126 (55.5)	123 (57.5)	134 (50.6)	47 (36.2)	
Stage IV	14 (8.5)	13 (5.7)	4 (1.9)	20 (7.6)	1 (0.8)	
**Weight-for-age Z-score(n,%)**						
Missing Data (Not in P-value)		1 (0.44)	1 (0.47)			
Median (Q1, Q3)	-2.6(-3.9,-1.6)	-2.0(-3.2,-0.9)	-2.1(-3.2,-1.1)	-2.0(-3.2,-0.8)	-1.7(-2.7,-0.6)	<0.0001
>-2	58 (35.15)	116 (51.33)	101 (47.42)	132 (49.81)	78 (59.54)	0.0006
-3 to -2	36 (21.82)	44 (19.47)	50 (23.47)	57 (21.51)	30 (22.90)	
<-3	71 (43.03)	66 (29.20)	62 (29.11)	76 (28.68)	23 (17.56)	

**Table 9:** Characteristics at ART initiation of children in Central Africa IeDEA in Rwanda by year of ART.

	2004–05 n = 115 (%)	2006–07 n = 204 (%)	2008–09 n = 171 (%)	2010–11 n = 140 (%)	2012–13 n = 127 (%)	*p*-Value
**Age (months) (n,%)**						
Median (IQR)	90.2 (56.1, 120)	89.2 (42.0, 119)	90.7 (47.1, 123)	71.3 (23.4, 116)	44.8 (12.7, 105)	<0.0001
0–24	8 (7.0)	29 (14.2)	23 (13.4)	36 (25.7)	45 (35.4)	<0.0001
25–60	22 (19.1)	45 (22.1)	32 (18.7)	26 (18.6)	25 (19.7)	
61–84	21 (18.3)	26 (12.7)	20 (11.7)	12 (8.6)	11 (8.7)	
85–120	35 (30.4)	56 (27.4)	48 (28.1)	34 (24.3)	22 (17.3)	
121–156	29 (25.2)	48 (23.5)	48 (28.1)	32 (22.9)	24 (18.9)	
**Sex (n,%)**						
Male	56 (48.7)	114 (55.9)	94 (55.0)	69 (49.3)	61 (48.0)	0.47
Female	59 (51.3)	90 (44.1)	77 (45.0)	71 (50.7)	66 (52.0)	
**CD4 count (n,%)**						
Missing Data (Not in P-value)	15 (13.0)	21 (10.3)	15 (8.8)	17 (12.1)	26 (20.5)	
Median (Q1,Q3)	263(167,441)	385(234,821)	350(264,779)	526(324,1033)	579(305,1310)	<0.0001
<200	35 (35.00)	35 (19.13)	20 (12.82)	11 (8.94)	12 (11.88)	<0.0001
200–349	33 (33.00)	47 (25.68)	58 (37.18)	28 (22.76)	28 (27.72)	
350–499	13 (13.00)	24 (13.11)	18 (11.54)	20 (16.26)	7 (6.93)	
>500	19 (19.00)	77 (42.08)	60 (38.46)	64 (52.03)	54 (53.47)	
Median CD4 for 0–24 months	1087(233,1332)	1146(993,1784)	1207(1106,1854)	1479(894,1983)	1504(1010,2219)	0.3412
Median CD4 for 25–60 months	469(262,698)	625(452,874)	749(458,1064)	1113(634,1532)	1275(782,2178)	0.0001
Median CD4 for 61–84 months	325(167,441)	500(280,751)	447(282,698)	548(381,694)	481(251,801)	0.236
Median CD4 for 85–120 months	212(124,314)	295(167,407)	304(238,362)	355(295,433)	332(205,369)	0.0034
Median CD4 for 121–156 months	216(98.0,293)	252(183,381)	283(236,352)	336(254,536)	334(282,391)	0.0041
n for 0–24 months	8	27	21	28	28	
n for 25–60 months	19	37	29	23	19	
n for 61–84 months	19	24	19	12	11	
n for 85–120 months	31	51	44	29	21	
n for 121–156 months	23	44	43	31	22	
**WHO clinical stage (n,%)**						
Missing Data (Not in P-value)	68 (59.1)	71 (34.8)	54 (31.6)	43 (30.7)	44 (34.7)	<0.0001
Stage I	8 (17.0)	17 (12.8)	33 (28.2)	31 (32.0)	41 (49.4)	
Stage II	13 (27.7)	31 (23.3)	26 (22.2)	23 (23.7)	25 (30.1)	
Stage III	21 (44.7)	80 (60.2)	50 (42.7)	33 (34.0)	16 (19.3)	
Stage IV	5 (10.6)	5 (3.8)	8 (6.8)	10 (10.3)	1 (1.2)	
**Weight-for-age Z-score(n,%)**						
Missing Data (Not in P-value)	71 (61.7)	87 (42.7)	41 (24.0)	32 (22.9)	17 (13.4)	<0.0001
Median (Q1, Q3)	-2.6(-3.3,-1.7)	-2.0(-3.1,-0.9)	-1.8(-2.6,-0.7)	-1.3(-2.4,-0.5)	-1.3(-2.3,-0.2)	<0.0001
>-2	12 (27.3)	60 (51.3)	71 (54.6)	73 (67.6)	76 (69.1)	
-3 to -2	17 (38.6)	26 (22.2)	32 (24.6)	21 (19.4)	21 (19.1)	
<-3	15 (34.1)	31 (26.5)	27 (20.8)	14 (13.0)	13 (11.8)	

**Fig 1:**
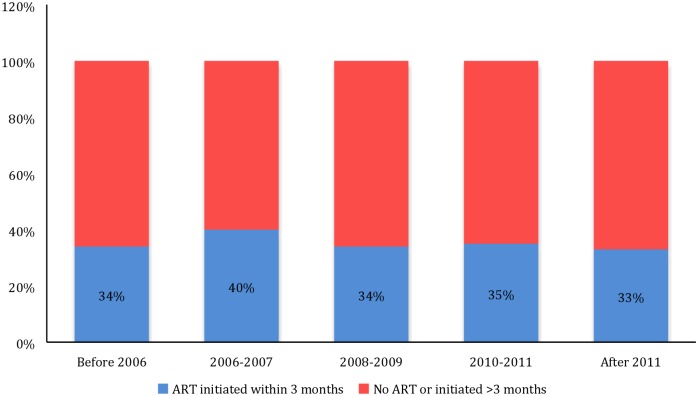
Proportion of children initiating ART within 3 months.

**Fig 2:**
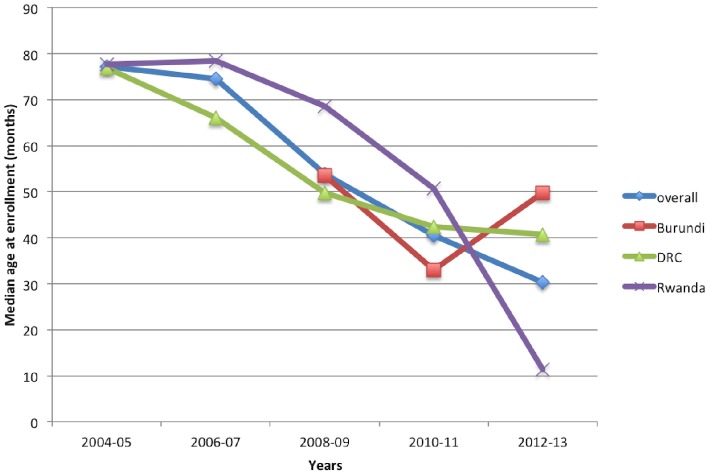
Median age at enrollment in HIV care.

**Fig 3:**
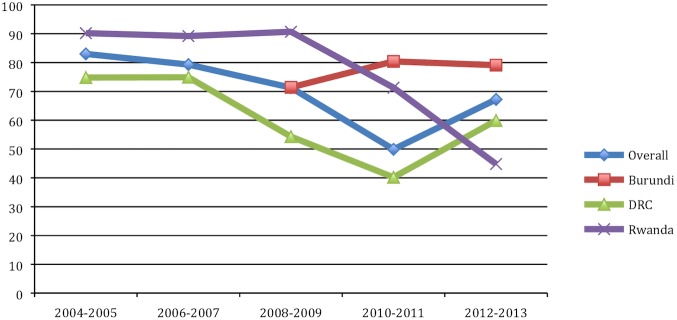
Median age at ART initiation.

**Fig 4:**
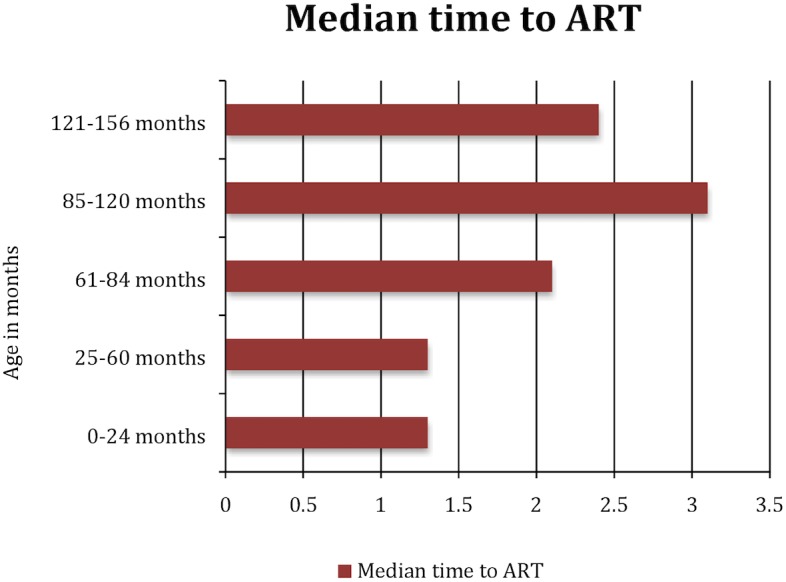
Median time from enrollment in care to ART initiation by age group.

Fig 1 depicts the trend in the proportion of children initiated on ART within three months of their enrollment in care. Before 2006, 34% of children were initiated on ART within three months of their enrollment into care. The proportion increased slightly to 40% in 2006–07 but remained fairly stable from 2008–13 at about one-third of children who enrolled in care. In Fig 2, we show the median age at enrollment into care in all countries from 2004-05-2012-13. Overall, age at enrollment into care continually declined in all countries until 2010–11. After 2011, age at enrollment increased in Burundi, plateaued in the DRC and continued to decline in Rwanda. As shown in Fig 3, overall median age at ART initiation declined until 2010–2011 and then rose slightly in 2012–2013. In Fig 4, we show the median time from enrollment in care to ART initiation among initiators by age group.

## Discussion

Early HIV diagnosis, enrollment in care, and ART initiation are critical to halt the rapid progression of HIV disease and reduce mortality in HIV-infected children [21]. Our study describes trends in characteristics of children enrolling in HIV care and initiating ART between 2004 and 2013 in the three Central African countries of Burundi, the DRC, and Rwanda to assess whether changes in the characteristics of children reflect changes in WHO guidelines for progressively earlier ART initiation.

Our results show that in all three countries the median age at enrollment in care (a proxy for HIV diagnosis) substantially decreased from 2004 to 2013, driven mainly by the increasing proportion of children ≤24 months of age, with a substantial increase observed in Rwanda and smaller increases in the DRC and Burundi. In Rwanda, for example, this increase reflects expanded access to DNA PCR for early infant diagnosis (EID) and the resulting increase in population coverage of EID from 28% to 72% from 2008–2011 after the country integrated EID with vaccination programs and invested in a robust mobile phone reporting system [22]. It is equally likely that increases in Rwanda reflect the effectiveness of the country’s ART program, considered one of the most effective for adults in the world also applies to children due to the availability of resources through PEPFAR, the Global Fund and others.

Along with the increasing proportion of children who were ≤24 months of age at enrollment, the median CD4 count at entry into care also increased over time from 563 in 2004–05 to 660 cells/μl in 2012–13, with similar increases in median CD4 count at ART initiation (310 in 2004–05 to 589 cells/μl in 2012–13). Further, the median CD4 count for all age groups increased at both enrolment into care and at ART initiation from 2004-05-2012-13 as shown in Tables 2 and 3. These results suggest that children 0–24 months and those 25–60 months were enrolling in care or initiating ART with higher CD4 cell counts compared with older children who enrolled in care or initiated ART with lower CD4 cell counts.

The likelihood of rapid progression of HIV disease and death among perinatally-infected children, and the evidence that early initiation of ART substantially reduces morbidity and mortality [8] led to the WHO’s 2010 recommendation for ART in all children <12 months regardless of CD4 count or clinical stage [9]. Our results show a significant decrease (p = <0.0001) in the median age at both enrollment in care and at ART initiation. Although some progress can be inferred on the basis of the results presented here, for example the falling median age at enrollment and ART initiation, there remains a substantial gap between the proportions of children <24 months who enrolled in care and those who initiated ART. Nonetheless, based on our analysis, we believe that the increase in CD4 count is due to children enrolling at younger ages versus being less sick at enrollment in care.

With regard to the number of children initiating ART, we observed that the number of children initiating ART in our sample is lower than those reported by Tene, et. al. [28] among children in Rwanda. The lower number of children initiating ART in our sample may be due to the selection of the clinics in our sample, which may not adequately represent the general population of pediatric HIV cohort, especially in Rwanda or may in fact reflect the proportion of children who are likely deceased or were lost to follow up and therefore unable to initiate ART. While this lack of representative sample is a limitation of our study, the findings presented are nonetheless critically important to highlight how changing guidelines are impacting on enrollment in care and initiation of ART among pediatric cohort in the sites where we obtained data.

While in-depth analyses are needed to fully understand the reasons for this gap, it is likely due to numerous structural factors, including time between sample collection and return of DNA PCR results, which is done centrally in all countries. Similarly, recent evidence from Zimbabwe showed that the lack of training in pediatric HIV among primary care providers is a strong barrier to successful implementation of comprehensive quality HIV service for children in extremely resource-poor settings like Central Africa [23]

The proportion of children without WHO staging data at both enrollment in care and ART initiation declined over time, it is plausible but hard to know how this could have biased our findings and/or in what direction although our focus in reporting the lack of WHO staging data was to highlight the importance of WHO staging in determining at what stage of disease classification children were enrolling in care or initiating ART. We note this as a limitation of the study.

Malnutrition, weight loss and failure to thrive are important clinical features in children presenting with AIDS and have been identified as causes of morbidity and mortality [24, 25]. Consistent with these studies [24–27], our median WAZ results suggest that many children in the cohort are either moderately or severely underweight, an indication of advanced disease, especially in the DRC where over half of children who enrolled in care through 2010–11 have a WAZ <-2. Again, this suggests that children who are considerably underweight when they enrolled in care or initiated ART may be presenting with advanced stages of the disease.

To our knowledge, this is the first study in Central Africa that combines data from 3 countries to examine trends in demographic and clinical characteristics of children at HIV care enrollment and at ART initiation. A strength of our analysis is that it included Rwanda, a country with robust data at the forefront of scaling HIV services and high retention rates among pediatric HIV patients [28], as well as Burundi and the DRC, two countries that consistently lag behind in pediatric HIV treatment and from which there are limited data. It is worth noting the different sample sizes, characteristics and non-representativeness of selected clinics in our sample. The participating clinics in CA-IeDEA countries are not a representative sample of clinics from those countries, and may include those with more technical capacity. In addition, our analyses are purely descriptive and in the absence of any strong evidence, it is difficult to attribute causation or to any other outcomes such as loss to follow up, death and transfers or to state categorically that the changes we observed are in response to policy changes. We have only recently begun to collect data on these outcomes, thus a limitation of our analysis is the inability to explore causal relationships behind the observed trends or directly test the effect of changing guidelines. Nevertheless, it seems highly plausible that the changes we observed may be a response to changes in guidelines even though other factors, such as increased donor funding and health system response to pediatric HIV care may have contributed to the observed changes. It is worth stating that in-depth analyses are needed to identify barriers to timely HIV diagnosis, enrollment into care, and ART initiation among children in the region. This is critical because Central and West Africa have the highest under five mortality worldwide [29], and further analyses are needed to examine the trends in outcomes (lost-to-follow-up, mortality) among HIV-infected children enrolled in care in this region.

## Conclusion

The proportion of children 24 months of age or younger at enrollment into HIV care in CA-IeDEA participating clinics has increased since 2004. However, this increase has not fully translated into early initiation of ART. Further analyses are needed to identify barriers to timely initiation of ART among children in HIV care.
